# Positive affect, depressive symptoms, and arthritis pain of elderly people over time

**DOI:** 10.12715/har.2015.4.16

**Published:** 2015-02-23

**Authors:** Jeong E. Lee, Eva Kahana, Boaz Kahana, Kaitlyn Barnes

**Affiliations:** 1 Case Western Reserve University, Cleveland, Ohio, USA; 2 Cleveland State University, Cleveland, Ohio, USA

## Abstract

**Background:**

Older adults frequently experience physical symptoms of arthritis pain. We examined the dynamic change of arthritis pain and depressive symptoms over time. We also addressed the influence of time varying arthritis pain on depressive symptoms and positive affect among community dwelling older individuals.

**Methods:**

Analyses were based on data from 4 annual follow-ups in a sample of 299 elderly residents (*M*=83.78) of Florida retirement communities. We estimated a hierarchical growth curve model that related the effects of time varying pain and characteristics of participants such as age, gender, cognitive functioning, emotional support and health. Growth curve modeling was used to assess changes in emotional well-being as a function of arthritis pain over time.

**Results:**

We found that depressive symptoms increased over 4 years whereas positive affect declined over 4 years with significant between-person differences in levels and slopes. As predicted, changes in arthritis pain co-varied with both depressive symptoms and positive affect over time. Gender, cognitive functioning, health conditions and emotional support from others were associated with between person differences in level of emotional well-being.

**Conclusions:**

Our findings suggest that conceptualization of emotional well-being of older adults as a dynamic, changing construct applies both depressive symptoms and positive affect. Findings also suggest that arthritis pain as well as emotional support contribute to depressive symptoms and to positive affect among older adults with arthritis.

## Introduction

Arthritis pain often leads to many adverse outcomes. This commonly acknowledged fact has been well documented in many studies [1–3]. Our study examines the effect of arthritis pain on emotional well-being, such as positive affect and depressive symptoms, over time by conducting a longitudinal study that allows for distinguishing concurrent from chronic pain. We acknowledge the widely documented adverse effects of arthritis on depressive symptoms and inquire whether such influences also extend to the ability to experience positive affect in late life among older adults. We also place psychological well-being of arthritis patients in a broader context by exploring demographic characteristics and health as additional stress related antecedents of depressive symptoms and positive affect. Finally, we consider important social and psychological resources, such as emotional support and intact cognitive functioning, as they impact the two well-being outcomes under consideration.

Theoretical models are useful in explaining the linkages between arthritis and emotional well-being of older adults. The theory of the disability cascade put forth by Verbrugge and Jette [4] posits that physical disability and negative quality of life outcomes (e.g., depressive symptoms) often result from functional impairment, following natural sequences of disease related events. Formulations of the disablement process specific to arthritis (AR) [2] propose that disablement in AR starts from pathology that interrupts the normal bodily processes or structures. These interruptions often lead to worsening emotional well-being. Indeed, the linkage between arthritis pain and depressive symptoms has been consistently found, with co-occurrence rates of 30–50% [5–7]. Other empirical studies have also supported the findings that pain often precedes depressive symptoms among older adults [1,8]. Thus, the proposition that arthritis pain leads to more depressive symptoms in community-dwelling older persons has achieved considerable credibility. However, previous studies examining the physical consequences of arthritis pain typically utilized cross-sectional data, only measuring pain at one time. Consequently, prior studies could not distinguish transient states of arthritis pain from more chronic arthritis pain.

Although prior research indicates that arthritis pain precedes depressive symptoms [9–11] studies rarely examine the concurrent arthritis effect on positive affect among community elders who experience arthritis pain. Recently, scholars have begun to pay more attention to the role of positive affect as a source of resilience in adaptation from the life stressors or health conditions [12]. However, changes in intensity of arthritis pain may also have a negative impact on older adults’ positive affect over time, due to repeated episodes of pain and discomfort. Thus, it is possible that change in arthritis pain over time affects how older adults feel about themselves as well as their positive affect.

In addition to examining the effects of arthritis pain over time, we were interested in evaluating the influences of demographic characteristics (e.g., gender, age), health conditions, cognitive functioning and emotional support on emotional well-being. Health conditions are often associated with lower levels of positive affect and higher depressive symptoms [13]. Furthermore, better cognitive functioning represents an important psychological resource and is often associated with lower depressive symptoms and higher positive affect [14]. Previous research suggests that emotional support from friends and family offers needed psychological resources and has a powerful impact on mental health of community dwelling older adults who live with arthritis [7,15]. Given its known function in alleviating the negative impact of stressors, we hypothesize that emotional support from others will be associated with both higher positive affect and with lower depressive symptoms.

The study addresses the following questions: (1) among arthritis patients, do older adults’ depressive symptoms and positive affect change over time? (2) If there is a change, is the change related to older adults’ arthritis pain over time when accounting for baseline characteristics? Given prior studies on affective well-being of older adults, we first hypothesized that (a) older adults’ depressive symptoms will increase but positive affect will decrease over time and (b) there will be significant between-person differences in level and rates of change, both in depressive symptoms and positive affect. Second, we hypothesized that changes in emotional well-being will co-vary with (a) older adults’ arthritis pain over time, and (b) other baseline characteristics such as sociodemographic and psychosocial resources.

## Methods

### Sample

The data for this study were collected as part of a larger panel study which focused on late life adaptation of community-dwelling elderly individuals. Participants were selected from a large, age-segregated, older adult community located in central Florida, where residents live independently and provide for their own care [16, 17]. To be eligible for this study, participants had to be (1) age 72 years or older, (2) living in Florida at least 9 months of the year, and (3) healthy enough to complete a 90 minute face-to-face interview. The final sample for the first wave of the study comprised 1,000 respondents, who completed face-to-face interviews. As part of the longitudinal project, respondents were contacted and interviewed annually with face-to-face interviews. Death is the main source of attrition.

The current study used four years of data from Waves 8 to 11 of our panel study (*N*=299). These study waves were chosen because the measurement of arthritis pain and report of diagnosis of arthritis by a clinician was introduced in the 8^th^ study wave until 11^th^ wave. In the selected waves, participants were asked more specific questions regarding their arthritis pain and their functioning resulting from arthritis pain. Of the eligible 373 participants, we narrowed the sample down to 299 participants who reported that they had been diagnosed with arthritis by clinicians. At the time of Wave 8, the average age of participants was 83.78 (*SD*=4.55). There were 76 (25%) men and 223 (75%) women in the sample.

### Measures

#### Depressive symptoms

Depressive symptoms were assessed with a 10-item version of the Centers for Epidemiologic Studies—Depression scale (CES–D) [18], a widely used self-report depression measure in community studies. Shortened versions of the CES-D have been found to be internally reliable, valid, and highly correlated with the full scale version (*r* = 0.96) in previous studies [19, 20]. In this study, participants reported how often they experienced specific symptoms during the past week, on a scale from 1 (never/rarely) to 5 (all of the time). We summed the scores to indicate that higher scores reflected more depressive symptoms. Given that usual scoring of the CES-D is on a scale from 0 to 3; our shortened measure provided more variation in depressive symptoms. The average of the scores ranged from 19.57 to 21.10 (*SD* =5.76 – 6.43) across 4 years. Cronbach’s alpha ranged from .847 to .882.

#### Positive affect

Participants’ positive affect was assessed with a modified version of the Positive and Negative Affective Schedule (PANAS) [21]. Participants were asked to rate the extent to which they felt a series of different positive emotions in the past year. Investigators sometimes refer to scores obtained through this procedure as a “trait” affect [21]. Given that we are only using positive affect, we did not include negative affect items from the whole PANAS scale. Respondents rated their experience of emotions on a 5-point Likert Scale, ranging from 1 (not at all) to 5 (extremely). We summed the scores so that higher scores indicate higher positive affect. Chronbach’s alpha ranged from .84 to .89 across the 4 years.

#### Arthritis pain

Pain severity during the past month was assessed with the subscale of the Arthritis Impact Measurement Scale (AIMS2) [22]. Among the subscales, we included five items regarding the severity of pain as well as morning stiffness, and sleeplessness due to arthritis. Ratings were made on a 5-point Likert Scale ranging from 1 (none or no days) to 5 (severe or all days). The average of the scores ranged from 10.02 to 10.8(*SD*=4.96–5.6), and Cronbach’s alpha for this scale ranged from .84 to .87 across 4 years.

#### Emotional support

Emotional support was assessed with the support scale developed by the co-authors of the paper. Participants reported how much emotional support they received from spouse, family or friends/neighbors during the past year. A total of 12 questions addressed the following areas of emotional support: listening, providing understanding, showing concern, expressed affection towards the participant (4 items each for spouse, family and friends/neighbors). For example, participants were asked, "How emotionally supportive people have been to you in the past year?” Participants answered using a 5-point scale, ranging from 1 (*none*) to 5 (*very much*). We summed the scores for all types of emotional support, so a higher score means more emotional support received from spouse, family members and friends/neighbors. The Cronbach’s alpha for this scale ranged from .8 to .84 across 4 years.

### Control variables

Several measures that could affect the relationship between pain and depressive symptoms were considered. Demographic characteristics such as age, gender, health conditions, and education were included (Table 1). Chronic health conditions were measured by a modified version of the OARS [23] summing the prevalence of 20 health conditions, including heart trouble, cancer, and diabetes. Cognitive functioning was measured by the Short Portable Mental Status Questionnaire (SPMSQ) [24], which is often used to assess cognitive impairment in the elderly adults.

Prior to analyses, we estimated bivariate associations between potential control variables and dependent variables (i.e., positive affect and depressive symptoms). Control variables included: age, gender, education, and health problems. Inclusion of control variables that are not associated with a dependent variable may generate spurious associations. Given that the education of participants did not show a significant association with depressive symptoms or positive affect, education was not included in our analyses.

### Analysis

To analyze the change in depressive symptoms and positive affect over time, we utilized growth curve analysis techniques to fit a series of multilevel models for change in the longitudinal data for all participants. The growth curve models allow us to evaluate longitudinal data at two levels (1) at the intra-individual level and (2) at the inter-individual level [25,26]. Therefore, our aim is to utilize growth curve models to provide a better understanding of changes in emotional well-being at the intra-individual level.

First, we calculated an unconditional means, no growth model, with no predictors of older adults’ depressive symptoms and positive affect over time. This unconditional means model provides an unadjusted estimate of the level of depressive symptoms or positive affect across all participants and all waves of data collection. It also allowed us to determine how much variance in the older adults’ reports of depressive symptoms could be attributed to between person level and within person level variance. From this estimates, we calculated the intra class correlation coefficient. Tested models indicate that there was substantial within-person variation in depressive symptoms (ICC=.66; 66% variance between persons, 34% variance within persons) and less in positive affect (ICC=.63, 63% variance between persons, 37% variance within persons).

To address hypothesis 1 that there will be a change over time in older people’s depressive symptoms/positive affect and (b) significant between person variation over time in level and slope, we estimated a linear growth curve model of change entering time into the equation at level 1 (within-person level).

To address hypothesis 2 that the relationship between older adults’ arthritis pain over time with their change in depressive symptoms and positive affect, we split arthritis pain into the within person and between person variance components to account for mean levels and variation of level in older adult’s depressive symptoms over time separately.

We then entered the within person variance (change in arthritis pain) into the Level 1 model. Next, we added the mean level of older adults’ arthritis pain, demographic variables such as age, gender, their general health conditions and resource variables of cognitive functioning, and emotional support received from others. We first entered all the characteristic variable interaction with time. We present the trimmed, parsimonious model that only includes significant relationships. All the analyses were run using the MIXED procedure in SAS [27]. Goodness of fit indices (e.g., Deviance -2 log likelihood, Akaike Information Criterion, and Bayesian Information Criterion) facilitated model comparisons.

## Results

Table 1 displays the means and standard deviations among the key study variables over 4 years. We first present findings regarding changes in depressive symptoms and positive affect using growth curve modeling. Then, we present the models examining the co-variation between emotional well-being (i.e., depressive symptoms and positive affect) and arthritis pain. Next, we present the full trimmed model including demographic variables and baseline level independent variables and arthritis pain as a between person effect.

### Changes in depressive symptoms and positive affect

The first hypothesis predicted (a) significant change over time in older adult’s depressive symptoms and positive affect and (b) significant variation over time in level and slope of depressive symptoms and positive affect. The linear multilevel model with only time as a predictor indicates significant change over time in depressive symptoms and significant variation in slope and level (β=.62, *p*<.001; Model 1 in Table 2). That is, at any point, one month of time was associated with a .62 point increase in depressive symptoms. In addition, there is a significant amount of between person variance in the intercept and a significant amount of between person variance in slope. With regard to positive affect, time also had a significant effect on positive affect (β=.35, *p*<.001; See Model 1 in Table 3). In sum, we found a significant increase in slope for reports of depressive symptoms and positive affect among older adults over 4 years and significant variation in older adults’ reports of depressive symptoms and positive affect in both level and change over time.

### Time varying arthritis pain and depressive symptoms and positive affect

In addressing Hypothesis 2, that older adults’ depressive symptoms would significantly co-vary with their arthritis pain over time, we found that time remains a significant predictor when the model includes the within person variance (See Model 2 in Table 2). We then added demographic variables, emotional support and arthritis pain as a between person effect as well as their interaction with time and trimmed the model to be parsimonious (See Model 3 in Table 2).

Overall, the inclusion of covariates did not reduce the effect of change of arthritis pain on concurrent depressive symptoms (β=.11, *p*<.001). In addition, higher levels of emotional support from others and higher cognitive functioning are related to lower levels of initial depressive symptoms (β=−.26, −.43, respectively, *p*<.001 for all). Female participants reported higher levels of depressive symptoms than male participants (β=2.24, *p*<.001). Having higher baseline reports of health problems are related to higher initial reports of depressive symptoms (β=.31, *p*<.001). Finally, the effect of mean level Arthritis pain on depressive symptoms was .33 (p<.001), indicating that higher levels of arthritis were associated with higher levels of depressive symptoms. In sum, the test of Hypothesis 2 indicates that changes in older adults’ depressive symptoms co-varied with their Arthritis pain over time even after considering the significant effect of older adults’ gender, cognitive functioning, health conditions, and emotional support on the level of depressive symptoms.

With regard to positive affect, we took a similar approach. When we included the within person variance (split from the between person variance) of arthritis pain, time was a significant predictor of positive affect. Finally, all the covariates were added to next full model (See Model 3 in Table 3) to examine if the relationship between arthritis pain and positive affect could be explained by demographic variables, health conditions, cognitive functioning and emotional support. Here, we included a parsimonious model (by trimming non-significant interaction; See Model 3 in Table 3). Higher emotional support, and higher cognitive functioning predicted higher levels of positive affect (β=.41, 44, respectively, *p*<.01 for all). More health conditions significantly predicted lower levels of positive affect (β=−.12, *p*<.001). In sum, changes in positive affect also co-varied with their arthritis pain over time (β=−.08, *p*<.05), even after accounting for the significant effects of age, health, cognitive functioning and emotional support on the level of positive affect.

## Discussion

The purpose of this study was to build upon previous research linking arthritis pain and emotional well-being by using growth curve models to examine if there were significant changes in emotional well-being among older adults across time (4 years). Additionally, we examined whether participants’ age, gender, general health, cognitive functioning, and emotional support influence changes in emotional well-being among older adults. In general, the results of the growth curve models provided support for our hypotheses. We found within person variation in depressive symptoms and in positive affect. Interestingly, the older adults in our sample reported more depressive symptoms over time, but also reported increased positive affect over time. Given that most empirical studies found that depression in later life is a common experience and it persists over time [28], our finding confirms its persistence, but also demonstrates that it increases over time. As previously mentioned, our results revealed that positive affect also increased over time. This finding is consistent with socio-emotional selectivity theory [29], which posits that the role of affective well-being improves over the life course [30,31]. This finding also suggests that the conceptualization of emotional well-being of older adults as a dynamic and changing construct in late adulthood.

Our findings further demonstrate that changes in arthritis pain severity are related to emotional well-being of older adults. As predicted, we found an independent effect of within person changes in arthritis pain over time on depressive symptoms and positive affect. Our findings are in line with prior studies that show that pain often precedes the development of depression [8]. Moreover, this relation remains significant even when corrected for health and other social resources, which is consistent with earlier findings [32]. The results we found with respect to the effect of pain on positive affect also strengthen the finding of previous studies, as prior studies examining this association often look at the short term relation between positive affect and arthritis pain [33]. In our study, we included a 4 year follow up period assessed at 1 year intervals in a large group of community dwelling older people.

Few predictors were foretelling of initial level of emotional well-being of older adults. Consistent with prior research, having more emotional support was predictive of lower depressive symptoms and higher positive affect [34, 35]. In addition, higher cognitive functioning was associated with lower depressive symptoms and higher positive affect. Gender and health problems were also predictive of the initial level of emotional well-being of older adults. Interestingly, female participants reported higher levels of depressive symptoms and also higher positive affect. In addition, better cognitive functioning was associated with lower levels of depressive symptoms and higher positive affect. These findings are consistent with prior research documenting gender differences in depressive symptomatology [36, 37]. Our findings linking chronic illnesses to adverse mental health outcomes confirm expectations of the disability cascade formulation [4]. In conclusion, these findings underscore the need to examine the effect of arthritis pain when considering emotional well-being of older adults. In particular, older adult’s varying pain may affect their well-being over time. Second, these findings highlight the complexity of understanding the emotional experiences of older adults. There is still significant variance to be explained in both the intercept (level) and the slope (change over time). There may be additional characteristics of older adults and changing processes involving arthritis pain that trigger changes in emotional well-being of older adults. By understanding this process, we can consider strategies that will help older adults improve their well-being.

## Study limitations

These findings are based on a unique sample that represents both a strength and limitation of this study. The focus on long term study participants (8–11 years) yielded a hardy sample. These are older adults who not only survived to their mid-80s, but are still living independently in retirement communities that do not offer any services. It is possible or even likely that older adults with more compromised health trajectories would portray even greater declines in psychological well-being. Given this unique sample characteristics, we might expect that the relationships observed in this study may be conservative, with steeper declines likely in more impaired and disadvantaged groups of older adults. Second, the initial sample selection and subsequent attrition could have limited the representativeness of the study population and generalizability of findings. However, a large proportion of the losses to follow up are due to death. The sample drawn for this study included primarily Caucasian respondents with slightly higher than average income and education. Thus, the results may not be generalizable to other demographic groups. Third, despite the longitudinal nature of the study, conclusions about directionality of arthritis pain and emotional well-being of older adults remain unclear. We can only conclude that there is a time co-varying relationship. Furthermore, modeling the data with a different time matric, such as time since arthritis diagnosis may also reveal different perspectives on the processes we found here.

## Conclusions

The findings of our study are valuable from several perspectives. First, utilizing the growth curve model allowed us to build upon previous research by examining not only mean level change (fixed effects), but also intra-individual changes (random effects). We found changes both in depressive symptoms and positive affect both in mean level change as well as within person change over time. Second, the effect of arthritis pain on positive affect has not been extensively studied, particularly over a long period of time in a large number of individuals. Finally, our findings suggest that treatment of arthritis pain could not only affect elderly patients’ depressive symptoms but also contribute to positive emotional well-being.

## Figures and Tables

**Table 1:** Means and standard deviation of key variables (1 year apart)

Variable	T1	T2	T3	T4

	*M (SD)*	*M(SD)*	*M(SD)*	*M(SD)*
Age	83.78 (4.55)			
Education	12.52(2.54)			
Health problems	13.72(6.31)	13.93 (6.17)	14.53 (5.97)	15.36(6.61)
Cognitive functioning	9.22 (1.54)	9.17 (1.71)	9.18 (1.52)	8.74 (1.98)
Emotional Support	3.87 (1.7)	3.55 (1.56)	3.72 (1.58)	3.79 (1.62)
Arthritis Pain	10.2 (5.0)	10.02(4.96)	10.2 (5.3)	10.8 (5.6)
Positive Affect	16.23 (3.84)	16.89(3.85)	17.62(3.65)	18.12 (3.87)
Depressive symptoms	19.57 (5.73)	20.03 (6.1)	20.33 (6.14)	21.10 (6.51)

Total	*N*=299	*N*=263	*N*=224	*N*=219

**Table 2:** Arthritis pain and depressive symptoms over time

	Model 1		Model 2		Model 3	

Parameter	Beta	SE	Beta	SE	Beta	SE
Fixed Effects						
Intercept	18.81***	.34	18.84***	.34	17.71***	1.68
Time	.62***	.11	.60***	.11	.40***	.10
Pain_resid (within person)			.11**	.04	.11**	.04
Predictors of Intercept						
Pain_mean (between person)					.33****	.05
Age					.0000	.00
Gender					2.24***	.55
Health					.31***	.07
COG					−.43***	.09
EMO					−.26***	.05
Random Effects						
Residual intercepts	16.84***	3.14	17.42***	3.16	12.46***	3.11
Variance in change over time	.02*	.01	.06**	.02	.03*	.01
Covariance intercept, change over time,	1.81**	.6	1.70***	.59	.85	.87
Residual Variances,	12.59***	.71	12.43***	.69	12.11***	.85
Model Fit						
−2 LL	6058.1		6054.5		5935.0	
AIC	6064.1		6060.5		5943.1	
BIC	6075.9		6072.3		5958.8	

Note. N=299 participants. Model based up to four occasions of measurement nested within 299 participants. −2LL= −2 log likelihood, relative model fit statistics.

COG: Cognitive function, EMO: Emotional support

* *p*<.05,

** *p*<.01,

*** *p*<.001

**Table 3:** Arthritis pain and positive affect over time

	Model 1		Model 2		Model 3	

Parameter	Beta	SE	Beta	SE	Beta	SE
Fixed Effects						
Intercept	16.13***	1.18	16.11***	.23	6.63	1.17
Time	.35***	.06	.32***	.06	.35***	.05
Pain_resid (within person)			−.07**	.02	−.04*	.02
Predictors of Intercept						
Pain_mean (between person)					−.08*	.03
Age					−.001	.0007
Gender					.43***	.32
Health					−.12***	.04
COG					.44***	.06
EMO					.41***	.03
Random Effects						
Residual intercepts	9.34***	1.60	9.45***	1.61	6.3***	1.35
Variance in change over time	.21*	.09	.2*	.1	.41	.3
Covariance intercept, change over time,	.04*	.02	.04*	.02	.08	.13
Residual Variances,	5.13***	.36	5.10***	.36	4.95***	.35
Model Fit						
−2 LL	5154.6		5153.4		4969.0	
AIC	5162.6		5161.4		4977.3	
BIC	5178.3		5177.2		4993.0	

Note. N=299 participants. Model is based on up to four occasions of measurement nested within 299 participants. −2LL= −2 log likelihood, relative model fit statistics.

COG: Cognitive function, EMO: Emotional support

* *p*<.05,

** *p*<.01,

*** *p*<.001
